# Racial/Ethnic Disparities in Food Pantry Use and Barriers in Massachusetts during the First Year of the COVID-19 Pandemic

**DOI:** 10.3390/nu14122531

**Published:** 2022-06-18

**Authors:** James P. Marriott, Lauren Fiechtner, Nick W. Birk, Daniel Taitelbaum, Angela Odoms-Young, Norbert L. Wilson, Lauren A. Clay, Rachel M. Zack

**Affiliations:** 1 Business and Data Analytics Department, The Greater Boston Food Bank, Boston, MA 02118, USA; lfiechtner@partners.org (L.F.); nicolaswilliam96@gmail.com (N.W.B.); dtaitelbaum@gbfb.org (D.T.); rmzack@gmail.com (R.M.Z.); 2 Department of Gastroenterology and Nutrition, MassGeneral Hospital for Children, Boston, MA 02114, USA; 3 Division of Nutritional Sciences, College of Human Ecology, Cornell University, Ithaca, NY 14853, USA; odoms-young@cornell.edu; 4 Duke Divinity School, Duke University, Durham, NC 27708, USA; norbert.wilson@duke.edu; 5 Department of Emergency Health Services, University of Maryland Baltimore County, Baltimore, MD 21250, USA; lclay@umbc.edu

**Keywords:** food insecurity, food pantry, racial disparities, COVID-19

## Abstract

This study sought to describe racial disparities in food insecurity, food pantry use, and barriers to and experiences with food pantries during the first year of the COVID-19 pandemic. We surveyed 2928 adults in Massachusetts regarding food access in the year before and during the first year of the pandemic. Weighted multivariable logistic regression models assessed racial differences in barriers to and experiences with pantry use during the pandemic. Black and Latino adults experienced the highest prevalence of food insecurity and pantry use. Additionally, Black and Latino adults reported more barriers to, but less stigma around, pantry use compared to White adults. Latino adults were less likely to know about pantry hours/locations and encounter staff who spoke their language. Black and Latino adults were also more likely to find pantry hours/locations inconvenient and have difficulty with transportation. The COVID-19 pandemic resulted in increased food insecurity, and food access inequities persisted. Programmatic policies to improve pantry access in communities of color could include increasing the hours/days that pantries are open, increasing bilingual staff, providing transportation or delivery, and creating multilingual public awareness campaigns on how to locate pantries.

## 1. Introduction

Food insecurity, which was experienced by 10.9% of Americans in 2019, is defined as a household-level economic and social condition of limited or uncertain access to adequate food [1,2]. Due to the economic repercussions of the COVID-19 pandemic, food insecurity increased in the United States for Black and Latino, but not White, households between 2019 and 2020 [3]. Charitable and federal food assistance programs grew considerably in response to increased economic need due to ramifications of the pandemic. Charitable food assistance use increased by 50%, with one in five people using food pantries and other private food assistance programs in 2020 [4]. In the United States, hundreds of food banks provide food to tens of thousands of food pantries, which serve local communities [5]. Food pantries are operated by a variety of organizations, including non-profit organizations, faith-based organizations, and community centers. The charitable food system serves as an emergency resource for those in need of food. As an example of the increased reliance on the charitable food system during the pandemic, The Greater Boston Food Bank (GBFB) reported that the number of people who received food assistance through its food pantry network doubled between May 2019 and May 2020. GBFB also reported a 58% increase in total pounds of food that it distributed to food pantries from March to December 2020 compared to the same period in 2019, highlighting the increased demand for charitable food assistance due to the pandemic [6]. Additionally, federal food assistance increased through the allocation of an extra USD 1 billion investment for The Emergency Food Access Program (TEFAP) and a 15% increase in Supplemental Nutrition Assistance Program (SNAP) benefits [7,8].

Although evidence indicates that food assistance programs are effective at reducing food insecurity [9,10,11], a high prevalence of food insecurity in the United States persists, particularly among people of color. Food insecurity prevalence is estimated to be three times higher among non-Hispanic Black households and two times higher among Hispanic households compared to non-Hispanic White households [3]. This discrepancy may be due in part to barriers that prevent equal access to food assistance programs [12,13,14]. For example, a study among Latin American immigrants in Toronto found that barriers to food access were, in part, due to language barriers leading to limited awareness of community food resources and limited availability of culturally preferred foods [15]. Previously identified barriers to food pantry use include a lack of knowledge regarding program location, the belief that one’s need is not high enough to justify pantry use, lack of resources (e.g., time and cooking equipment), special dietary needs, and experiences of discrimination [16,17,18,19]. While stigma is also a frequently cited barrier to food pantry use [19,20], one study found that there is less stigma among the public toward food pantries than food pantry users expect [21]. It is important to fully recognize and reduce these barriers to equitably increase access and reduce disparities in pantry use and food access. However, there is a lack of research in understanding racial and ethnic differences regarding barriers to food pantry access at the state level, particularly during the COVID-19 pandemic. This study hopes to add to the literature how barriers to food pantry use vary by race and ethnicity, particularly in a time of crisis, e.g., the COVID-19 pandemic. Past research on barriers to food pantries has either focused on a specific racial/ethnic group or the impact of the pandemic, but rarely both.

This study sought to better understand: (1) racial and ethnic disparities in food insecurity prevalence and (2) barriers to and experiences with food pantry use in Massachusetts to improve equitable access to food pantries. We hypothesize that racial/ethnic minorities have a higher prevalence of food insecurity and face more barriers in using food pantries.

## 2. Materials and Methods

### 2.1. Study Design and Participants

The MA Statewide Food Access Survey was modified from a survey developed by the National Food Access and Covid Research Team (NFACT) [22]. We conducted a cross-sectional survey of adults living in Massachusetts in 2020. Participants were recruited by Qualtrics, a survey research firm, to complete an online survey on food access and food security through the Qualtrics Panels Project between 19 October 2020 and 6 January 2021. The Qualtrics Panels Project allows for demographic quotas so that survey respondents represent the demographic distribution of a population of interest [23].

**Figure A1:**
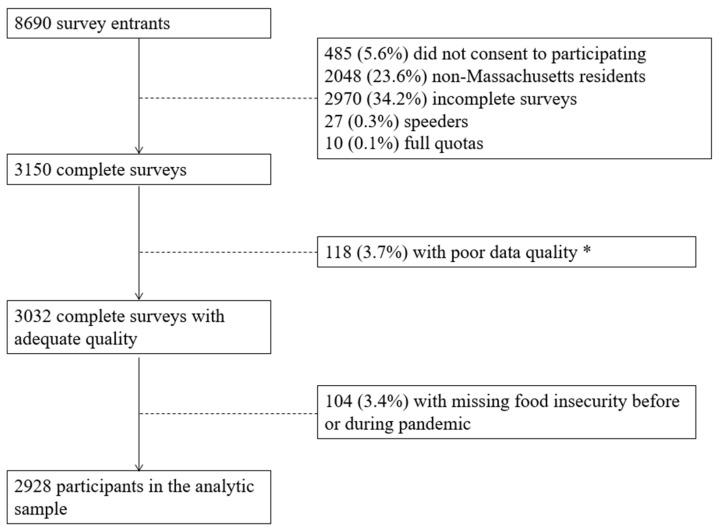
Study flow diagram. * Poor data quality was defined as having non-sensical free-text responses, age below 18 or above 100, survey response information which differed greatly from the Qualtrics demographic information, or more than two data flags (e.g., respondent’s age does not match household composition, reported diaper insecurity without children in household, use of school meals program or WIC without children in household, reported using food pantry but state none of their groceries come from pantry, reported using some form of pandemic food assistance generally but reported not using any specific food assistance program).

Adults aged 18 years or older, living in Massachusetts since at least 1 January 2020, and with the ability to complete the online survey in English or Spanish were eligible for participation. Low-income adults were oversampled (24% with household income <$25,000, 25% with income ≥$25,000 to <$50,000, 18% with income ≥$50,000 to <$75,000, 33% with income ≥$75,000) to obtain an adequate sample of those most likely in need of food assistance (Table A1). Of the 8690 survey entrants, 3150 (36%) completed the survey. The median survey completion time was 16 min. To eliminate those who sped through the survey, respondents who completed the survey more than two standard deviations below the median duration were excluded. A total of 118 responses were excluded due to poor data quality. Exclusion criteria are outlined in Appendix A. We further excluded 104 respondents with missing data on food security status for either the year before the pandemic or during the first year of the pandemic for a total of 2928 responses (Figure A1). The survey was offered in English and Spanish: 2907 respondents (99%) completed it in English, and 21 respondents (1%) completed it in Spanish.

### 2.2. Measures

The survey included questions on demographics, food assistance use, and facilitators and barriers to federal and charitable food assistance use. Many survey questions referred to reference periods of “the year before the pandemic” and “since the pandemic”. We defined the start of the COVID-19 pandemic as 11 March 2020, the day of the World Health Organization declaration [24]. We use “the year before the pandemic” to refer to March 2019–March 2020 and “since the pandemic”/“the first year of the pandemic” to refer to March 2020–January 2021.

Demographics: Participants were asked to self-identify their race. Due to small sample sizes in certain racial/ethnic groups, those who responded as Middle Eastern or North African (*n* = 16), American Indian or Alaskan Native (*n* = 35), Native Hawaiian or Pacific Islander (*n* = 1), or other/multiracial (*n* = 50) were categorized as “other” race. Hispanic/Latino was defined as any person identifying as Hispanic or Latino, regardless of belonging to any other racial or ethnic group.

Food insecurity: Food insecurity was measured using the USDA 6-item U.S. Household Food Security Survey Module with two reference periods: the year before the pandemic, which was recalled retrospectively, and the past 30 days [25].

Food pantry use: Respondents were asked whether they had used food pantries (yes/no) during the pandemic.

Barriers to and experiences with food pantry use: All participants were given six statements on potential barriers to food pantry use and asked to respond to each using a Likert Scale. Additionally, participants who reported food pantry use during the pandemic were given fifteen statements on their experiences with using food pantries and asked to respond to each using a Likert Scale. The response options were “Strongly agree”, “Agree”, “Disagree”, or “Strongly disagree”, which were dichotomized to a binary “Agree”/”Disagree” for analysis. 

### 2.3. Statistical Analysis

All analyses were restricted to those who had non-missing data on food security status before and during the pandemic. Analyses on barriers to food pantry use were restricted to adults who experienced food insecurity during the pandemic but did not use food pantries during the pandemic (*n* = 498). Analyses on experiences with food pantries were restricted to adults who used food pantries during the pandemic, regardless of food security status (*n* = 520). Participants with incomplete data on the food pantry barriers and/or experiences questions (*n* = 272) were excluded from analysis.

We used a raking procedure to generate sampling weights using the anesrake R package and trimmed weights greater than five. Weights were calculated using demographic distributions of gender, age group, race/ethnicity, education, income category, and geographic region for adults in Massachusetts obtained from American Community Survey (ACS) data from the United States Census Bureau website and the tidycensus R package [26]. The demographic distribution of the weighted MA Statewide Food Access Study data closely matched the ACS data, with the largest difference being that the MA Statewide Food Access Study weighted data had an overrepresentation of adults with children (Table A1).

For results to be representative of the Massachusetts adult population, all point estimates, confidence intervals, and hypothesis tests were calculated using the survey R package to account for the sampling weights. Differences in food insecurity, food pantry use, food pantry experiences, and food pantry barriers by racial/ethnic group were evaluated using χ^2^ tests. We used logistic regression to examine differences in food pantry barriers and experiences by racial/ethnic group. We adjusted for income category, presence of children in household, age group, education, and gender. Refer to Table A1 for covariate categories.

Analyses were conducted using R version 4.1.0 (R Core Team, Vienna, Austria). Statistical significance was defined as a 2-sided *p*-value less than 0.05.

## 3. Results

### 3.1. Food Insecurity

Results from the survey indicated that food insecurity in Massachusetts increased from 19% in the year before the COVID-19 pandemic (retrospectively reported) to 30% during the first year of the pandemic (Table 1). Food insecurity increased across all racial and ethnic groups, with Black and Latino adults experiencing a significantly higher prevalence of food insecurity both before and during the pandemic compared to White adults. Asian adults experienced food insecurity levels similar to those of White adults before and during the pandemic. Additionally, Black and Latino adults experienced the largest absolute differences in food insecurity prevalence among all racial/ethnic groups at 14%. Adults with children in the household had a higher prevalence of food insecurity before the pandemic compared to adults without children in the household (27% vs. 14%), as well as during the pandemic (42% vs. 22%). The absolute difference in food insecurity for adults with children was 15%, whereas this difference for adults without children was 8%. Of Latino adults with children, 64% reported being food-insecure during the pandemic, significantly higher than the 36% of White adults with children reporting food insecurity during the same time period.

### 3.2. Food Pantry Use

Similar to the trends seen in food insecurity prevalence, food pantry use in Massachusetts increased from 9% in the year before the pandemic to 12% during the pandemic (Table 2). Among both Asian and White adults, food pantry use increased from 6% to 9%. The prevalence of food pantry use among Black adults increased from 20% to 26% and from 21% to 28% among Latino adults. Overall, food pantry use for Black and Latino adults was significantly higher compared to White adults. Among individuals experiencing food insecurity, only 27% reported using a food pantry before the pandemic. This increased slightly to 32% during the pandemic, with most food-insecure adults still not using food pantries. Among those experiencing food insecurity during the pandemic, Latino adults reported significantly higher usage of food pantries (41%) compared to White adults (29%).

### 3.3. Barriers to Food Pantries

**Figure A2:**
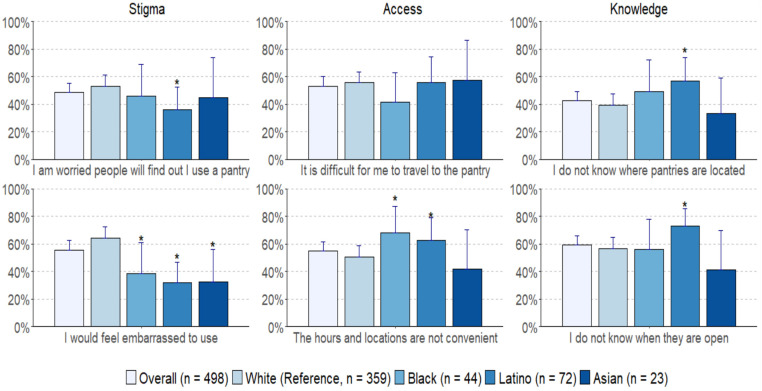
Barriers to food pantry use among food-insecure adults not using pantries during the COVID-19 pandemic. Chi-square tests were performed for each statement for each racial/ethnic group compared to the white reference group; * indicates a *p*-value < 0.05.

Barriers to food pantry use among those who were food-insecure but not using pantries were reported with the following frequencies: not knowing when pantries are open (59%), feeling embarrassed to use a pantry (56%), inconvenient hours/location (55%), difficulties with traveling to the pantry (53%), worried that others would find out they use the pantry (48%), and not knowing pantry locations (42%) (Table 3, Figure A2). Frequency of reported barriers differed by race/ethnicity. In total, 56% of Latino adults reported not knowing where food pantries were located compared to 39% of White adults (aOR 2.98; 95% CI 1.25–7.10). Additionally, 74% of Latino adults did not know when the pantries were open, whereas 57% of White adults expressed the same concern (aOR 2.82; 95% CI 1.23–6.45). Regarding access to food pantries, 76% of Black and 62% of Latino adults reported that the hours were not convenient compared to 51% of White adults (Black aOR 4.73, 95% CI 1.68–13.30; Latino aOR 3.61, 95% CI 1.53–8.50). For barriers related to stigma, White adults generally reported higher levels of stigma than people of color for using food pantries. A total of 65% of White adults indicated that they would feel embarrassed to use pantries, while 37% of Black (aOR 0.27, 95% CI 0.10–0.76), 32% of Latino (aOR 0.28, 95% CI 0.13–0.64), and 33% of Asian (aOR 0.23, 95% CI 0.08–0.63) adults indicated the same.

### 3.4. Experiences with Food Pantries

**Figure A3:**
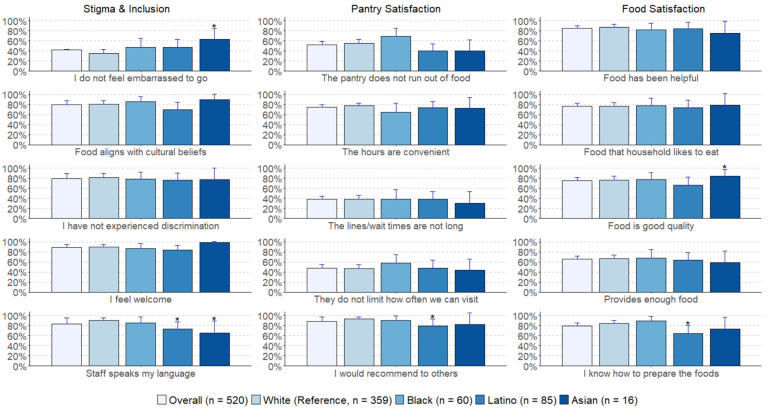
Experiences with food pantries among food-insecure adults during the COVID-19 pandemic. Chi-square tests were performed for each statement for each racial/ethnic group compared to the white reference group; * indicates a *p*-value < 0.05.

Regarding stigma and inclusion, the majority of pantry users reported feeling welcome (89%), encountering staff who speak their language (84%), receiving food that aligns with cultural beliefs (80%), and not experiencing discrimination (79%) (Table 4, Figure A3). With respect to pantry satisfaction, most pantry users would recommend food pantries to others (89%), reported the hours are convenient (74%), and that the pantry does not run out of food (52%). However, a minority of pantry users found that the lines/wait times are not long (38%). Regarding food satisfaction, pantry users agreed that the food has been helpful (85%), they know how to prepare foods (79%), pantries provide food that their household likes to eat (77%), the food is good quality (75%), and pantries provide enough food (66%).

Experiences with food pantries among pantry users during the COVID-19 pandemic were similarly analyzed using multivariable logistic regressions to identify racial/ethnic differences in food pantry experiences. Only 73% of Latino and 65% of Asian adults reported encountering food pantry staff who speak their language, which is significantly less than the 90% of White adults reporting the same (Latino aOR 0.21, 95% CI 0.08–0.57; Asian aOR 0.09, 95% CI 0.02–0.57). Latino adults were also significantly less likely to recommend food pantries to others, with only 79% agreeing compared to 93% of White adults (aOR 0.29, 95% CI 0.10–0.88). In total, 84% of Asian adults agreed that the food at pantries was good-quality, which was significantly higher than the 76% of White adults who agreed with that statement (aOR 8.85, 95% CI 1.45–53.90).

## 4. Discussion

In this representative survey of Massachusetts residents, we observed an increase in food insecurity and food pantry use during the first year of the COVID-19 pandemic. These measures varied by race and ethnicity, with Black and Latino adults experiencing a significantly higher prevalence of food insecurity and food pantry use both in the year before and in the first year of the pandemic compared to White adults. Nearly one-third of food-insecure adults were not using food pantries, although use slightly increased during the pandemic. Among food-insecure individuals during the pandemic, certain barriers to food pantry use varied by race and ethnicity. Latino adults reported less knowledge regarding when pantries were open and where they were located; Black and Latino adults reported more difficulty accessing pantries in terms of transportation barriers and convenient locations/hours; and White adults reported higher levels of stigma surrounding food pantry use. Experiences with food pantries among pantry users during the COVID-19 pandemic also varied by race and ethnicity. Latino and Asian adults were less likely to encounter food pantry staff who spoke their language; Latino adults were less likely to recommend food pantries to others; and Asian adults were more likely to agree that the food is of good quality than White adults.

The increases that we find in food insecurity and food pantry use are consistent with other Massachusetts and nationwide surveys conducted during the COVID-19 pandemic [27,28,29]. Conversely, findings from the US Department of Agriculture (USDA) suggest that food insecurity in the United States remained stable from 2019 to 2020 [3]. Our findings may differ for several reasons. The results from the USDA are calculated from the Current Population Survey (CPS), which was conducted by telephone during the pandemic. Survey respondents may be less likely to be truthful during a telephone interview rather than an anonymous online survey due to stigma [30]. Additionally, the estimates in the present study may be slightly overestimated due to the weighted sample having a higher prevalence of households with children compared to the ACS data (Table A1). Since households with children are more likely to face food insecurity than households without children [3], this discrepancy may skew the estimates toward a higher percentage of the overall sample being food-insecure.

Racial disparities in food insecurity have been documented for many years and throughout different economic crises [14,31]. Structural racism contributes to and reinforces the disparately negative effects of food insecurity, which have been exacerbated by COVID-19 [32,33,34]. A study conducted in South Carolina with Black households found that a one-unit increase in a lifetime racial discrimination score was associated with a 5% increase in severe food insecurity when controlling for socioeconomic and demographic factors [35]. Although we find higher use of food pantries among Black and Latino adults compared to White adults, the present study shows that the disparities in food insecurity have persisted during the COVID-19 pandemic, with Black and Latino adults significantly more likely to experience food insecurity. The reasons for this are likely varied and complex. The racial differences in barriers to and experiences with food pantry use described in this study may be rooted in factors associated with poverty (e.g., transportation difficulties, childcare costs, and limited control over work schedules), which disproportionately impacts people of color. This in turn may hinder access to food pantries and contribute to these persistent racial disparities in food security. One study suggested that the concrete barriers to food pantry use, such as poor food quality and long lines, have different cultural interpretations [36]. For example, it found that concerns around long lines were more related to having to stand next to people deemed to be of lesser moral quality than the actual process of waiting a long time in line. The authors emphasized that understanding cultural differences in barriers and perceptions of barriers is crucial for fully understanding how to help people overcome these barriers.

Food banks and pantries could work towards alleviating these barriers by having additional or more varied hours of operation, particularly outside of standard work hours, providing home delivery or transportation, and engaging with people in their community with lived experiences to best understand how to provide food access in a culturally sensitive and convenient way. Additionally, while the responses to the stigma-related questions asked in this survey indicate that White people feel more stigma around using food pantries than people of color, an alternative interpretation is that historically marginalized groups are more open to support or have experience in accessing support more often than their White counterparts. Racial differences in stigma around food assistance have been previously noted. Welfare stigma has been associated with major depressive disorder in White, middle-aged, male, and able-bodied SNAP users, which suggests a deleterious effect of welfare stigma among those who are more likely to feel stigma due to stereotypical societal expectations [37]. These race-based differences regarding stigma towards food pantry use warrant further research to better understand why these discrepancies exist and how to combat them.

### Limitations and Strengths

While our survey design allowed for a representative sample of Massachusetts adults, this limited the sample size of certain racial groups, particularly Asian adults and other racial groups not described in this analysis, due to the relative racial/ethnic homogeneity in Massachusetts, where 71.1% of the population are non-Hispanic/non-Latino White [38]. The quota sampling approach we employed to obtain a representative sample is not a probability-based sampling method, which may introduce selection bias. While we weighted the sample on certain demographic variables to be representative of the Massachusetts population to account for this potential bias, there may be unknown factors associated with survey completion that we did not account for or have information about when conducting the weighting procedure. As the survey was available only in English and Spanish, this likely resulted in underreporting certain barriers to and experiences with food pantries, such as encountering staff who speak their language and receiving food that is aligned with cultural beliefs. Additionally, the survey was only offered online and therefore was not available to those without access to computers or smartphones. However, a Pew survey of Americans in 2018 found high rates of internet access (89% of non-Hispanic White, 88% of Hispanic, and 87% of Black Americans), even among low-income populations (81% among households reporting incomes of $30,000 or less) [39]. Given the ongoing pandemic, using an online survey reduced health risks for both study participants and research staff while supporting timely data collection. A further limitation is that the survey required participants to recall food insecurity and food pantry use in the year prior to the COVID-19 pandemic, which introduces the possibility of issues with recall. We did not gain a full understanding of all potential barriers to pantry use because we had a pre-defined list of barriers for respondents to choose from. Strengths of the study include a large representative sample of the Massachusetts population and detailed data on food pantry barriers and experiences. Additionally, this cross-sectional survey provided an efficient way to obtain data to address the impact of COVID-19 on food insecurity and food access in a timely manner given the urgency of the situation. It also allowed us to assess several aspects related to hunger, such as food insecurity and food pantry barriers.

## 5. Conclusions

Any increases in food insecurity are worrisome as the repercussions can extend beyond hunger. Food insecurity has been found to increase the risk of negative health outcomes, including diabetes, hypertension, poorer mental health, and higher healthcare costs [40,41,42]. While we found an increase in the prevalence of food insecurity during the first year of the pandemic, our findings also raise concerns for the long-term negative health outcomes related to food insecurity and hunger, particularly among communities of color.

Many programs and policies can be enacted at the community, state, and federal level to support equitable anti-hunger efforts. Community-based efforts can work to raise public awareness to reduce stigma surrounding food insecurity and share existing resources for food and financial assistance programs. Additionally, these efforts can engage with or be led from within communities of color to amplify voices of diverse individuals, increase access to bilingual staff and volunteers at food pantries, ensure that all people are welcome, reinforce that no photo identification is needed at pantries, and train anti-hunger organization staff and volunteers on recognizing implicit bias and maintaining the dignity of those requiring assistance. Efforts at the state and federal level should continue to prioritize funding as well as extend food and financial assistance policies and infrastructure investments to equitably reduce food insecurity and poverty. Policies aimed at reducing the burden of unemployment, general poverty, and income inequality will also be key in ensuring equitable hunger relief and economic recovery from the pandemic [43]. Further, as those who were already vulnerable to food insecurity were particularly impacted by the pandemic [44], poverty-reducing measures could work to reduce the burden on the charitable food system during times of widespread economic hardship [45].

Given the cross-sectional nature of this study, the data represent one snapshot of food insecurity and barriers to food pantry use during the first year of the COVID-19 pandemic. As a result, future research should continue to assess the dynamic and inequitable effects of the pandemic on food insecurity and food assistance while also evaluating the impact of the new and expanded anti-hunger programs and policies at the community, state, and federal level. Studies that assess the effect of charitable and governmental responses during the pandemic on anti-hunger efforts will allow these agencies to be better equipped to respond in the event of a similar crisis. Additionally, research that explores the role of racialized stigma would be greatly beneficial to understand why certain barriers to food pantry exist for certain groups and how to best ameliorate them.

## Figures and Tables

**Table 1:** Food insecurity prevalence before and in the first year of the pandemic overall and among those with and without children in the household by race/ethnicity.

Food Insecurity ^1^	Race/Ethnicity	*n* ^2^	Before Pandemic ^3^		First Year of Pandemic ^4^		Absolute Difference
			Prevalence (95% CI)	*p*-Value	Prevalence (95% CI)	*p*-Value	
Overall	Overall	2826	19% (17–21%)		30% (27%–32%)		11%
	White	2184	15% (13–17%)	Ref	24% (22%–27%)	Ref	9%
	Black	200	31% (22–40%)	**<0.001**	45% (35%–55%)	**<0.001**	14%
	Latino	292	44% (35–53%)	**<0.001**	58% (49%–67%)	**<0.001**	14%
	Asian	150	16% (8–23%)	0.823	26% (17%–36%)	0.660	11%
Adults with children in the household	Overall	1052	27% (23–31%)		42% (38%–47%)		15%
	White	717	21% (17–25%)	Ref	36% (31%–42%)	Ref	15%
	Black	113	36% (23–48%)	0.244	46% (32%–60%)	0.343	10%
	Latino	171	47% (36–59%)	**<0.001**	64% (53%–75%)	**0.015**	16%
	Asian	51	19% (7–31%)	0.834	30% (15%–45%)	0.882	11%
Adults without children in the household	Overall	1774	14% (12–17%)		22% (20%–25%)		8%
	White	1467	12% (10–14%)	Ref	19% (16%–22%)	Ref	7%
	Black	87	24% (12–36%)	**<0.001**	43% (28%–58%)	**<0.001**	19%
	Latino	121	37% (25–50%)	**<0.001**	48% (34%–61%)	**<0.001**	10%
	Asian	99	13% (3–23%)	0.915	24% (12%–36%)	0.733	11%

Chi-square tests with a *p*-value < 0.05 are bolded. ^1^ Food insecurity measured using the USDA 6-item U.S. Household Food Security Survey Module. ^2^ “Other” race/ethnicity (Middle Eastern or North African, American Indian or Alaskan Native, Native Hawaiian or Pacific Islander, multiracial, or other) not included in analysis; *n* = 102. ^3^ Before pandemic period defined as year before pandemic. ^4^ First year of pandemic period defined as after the WHO declaration of the COVID-19 pandemic on 11 March 2020 through the end of survey collection.

**Table 2:** Prevalence of food pantry use before and in the first year of the pandemic overall and among those experiencing food insecurity by race/ethnicity.

Food Pantry Use	Race/Ethnicity	Before Pandemic ^2^			First Year of Pandemic ^3^			Absolute Difference
		*n* ^1^	Prevalence (95% CI)	*p*-Value	*n* ^1^	Prevalence (95% CI)	*p*-Value	
Overall	Overall	2826	9% (7–10%)		2826	12% (11%–14%)		4%
	White	2184	6% (5–7%)	Ref	2184	9% (7%–10%)	Ref	3%
	Black	200	20% (13–28%)	**<0.001**	200	26% (17%–34%)	**<0.001**	5%
	Latino	292	21% (14–28%)	**<0.001**	292	28% (20%–35%)	**<0.001**	7%
	Asian	150	6% (2–11%)	0.800	150	9% (3%–15%)	0.946	2%
Adults with food insecurity	Overall	825	27% (22–32%)		1188	32% (28%–36%)		5%
	White	570	24% (18–29%)	Ref	845	29% (24%–34%)	Ref	5%
	Black	82	32% (18–46%)	0.252	105	40% (26%–54%)	0.123	8%
	Latino	142	32% (20–43%)	0.186	186	41% (30%–52%)	**0.042**	9%
	Asian	31	24% (1–47%)	0.962	52	25% (7%–43%)	0.696	1%

Chi-square tests with a *p*-value < 0.05 are bolded. ^1^ “Other” (Middle Eastern or North African, American Indian or Alaskan Native, Native Hawaiian or Pacific Islander, multiracial, or other) race/ethnicity not included in analysis; *n* = 102. ^2^ Before pandemic period defined as year before pandemic. ^3^ First year of pandemic period defined as after the WHO declaration of the COVID-19 pandemic on 11 March 2020 through the end of survey collection.

**Table 3:** Crude and adjusted odds ratios for statements pertaining to food pantry barriers by race, *n* = 498.

Domain	Statement	Race ^1^	% Agree ^2^	Unadjusted Odds Ratio (95% CI)	*p*-Value	Adjusted Odds Ratio (95% CI)	*p*-Value
Knowledge	I do not know where the pantries are located	Overall	42%				
		White	39%	Ref		Ref	
		Black	50%	1.55 (0.55–4.35)	0.402	1.77 (0.64–3.84)	0.274
		Latino	56%	2.31 (1.06–5.07)	**0.036**	2.98 (1.25–7.10)	**0.014**
		Asian	33%	0.80 (0.24–2.62)	0.708	0.76 (0.24–2.35)	0.629
	I do not know when they are open	Overall	59%				
		White	57%	Ref		Ref	
		Black	63%	1.43 (0.54–3.75)	0.469	1.40 (0.52–3.81)	0.507
		Latino	74%	2.24 (1.07–4.68)	**0.032**	2.82 (1.23–6.45)	**0.014**
		Asian	42%	0.62 (0.18–2.08)	0.436	0.59 (0.19–1.81)	0.354
Access	The hours and locations are not convenient	Overall	55%				
		White	51%	Ref		Ref	
		Black	76%	3.67 (1.40–9.62)	**0.008**	4.73 (1.68–13.30)	**0.003**
		Latino	62%	2.17 (1.00–4.70)	**0.051**	3.61 (1.53–8.50)	**0.003**
		Asian	42%	0.80 (0.24–2.69)	0.716	0.90 (0.32–2.56)	0.848
	It is difficult for me to travel to the pantry	Overall	53%				
		White	56%	Ref		Ref	
		Black	34%	0.44 (0.17–1.10)	0.078	0.50 (0.22–1.18)	0.113
		Latino	55%	0.91 (0.39–2.10)	0.826	0.84 (0.37–1.93)	0.687
		Asian	57%	1.10 (0.32–3.72)	0.880	1.26 (0.35–4.54)	0.720
Stigma	I am worried people will find out I use the pantry	Overall	48%				
		White	53%	Ref		Ref	
		Black	40%	0.62 (0.22–1.70)	0.349	0.42 (0.14–1.27)	0.124
		Latino	36%	0.47 (0.21–1.03)	0.058	0.49 (0.21–1.11)	0.086
		Asian	45	0.78 (0.23–2.62)	0.683	0.58 (0.18–1.51)	0.233
	I would feel embarrassed to use	Overall	56%				
		White	65%	Ref		Ref	
		Black	37%	0.34 (0.12–0.96)	**0.041**	0.27 (0.10–0.76)	**0.013**
		Latino	32%	0.23 (0.11–0.48)	**<0.001**	0.28 (0.13–0.64)	**0.002**
		Asian	33%	0.27 (0.09–0.84)	**0.024**	0.23 (0.08–0.63)	**0.004**

Notes: The sample is restricted to those who report being food-insecure during the COVID-19 pandemic, but do not report using a food pantry. Models were adjusted for income, presence of children in household, gender, age, and education. Odds ratios with a *p*-value < 0.05 are bolded. ^1^ Sample sizes: Overall = 498; White = 359; Black = 44; Latino = 72; Asian = 23; Incomplete responses = 243. ^2^ % Agree is defined as the percentage of those selecting “Strongly Agree” or “Agree”.

**Table 4:** Crude and adjusted odds ratios for statements pertaining to food pantry experiences by race, *n* = 520.

Domain	Statement	Race ^1^	% Agree ^2^	Unadjusted Odds Ratio (95% CI)	*p*-Value	Adjusted Odds Ratio (95% CI)	*p*-Value
Stigma and Inclusion	Food aligns with cultural beliefs	Overall	80%				
		White	81%	Ref		Ref	
		Black	85%	1.35 (0.52–3.51)	0.531	1.83 (0.67–5.00)	0.241
		Latino	69%	0.53 (0.23–1.25)	0.145	0.78 (0.32–1.91)	0.592
		Asian	89%	2.34 (0.31–17.80)	0.410	1.95 (0.30–12.90)	0.488
	Staff speaks my language	Overall	84%				
		White	90%	Ref		Ref	
		Black	85%	0.63 (0.20–1.94)	0.417	0.58 (0.20–1.71)	0.323
		Latino	73%	0.30 (0.12–0.76)	**0.011**	0.21 (0.08–0.57)	**0.002**
		Asian	65%	0.08 (0.02–0.36)	**0.001**	0.09 (0.02–0.57)	**0.010**
	I have not experienced discrimination	Overall	79%				
		White	81%	Ref		Ref	
		Black	78%	0.82 (0.32–2.10)	0.678	0.94 (0.30–2.95)	0.920
		Latino	76%	0.73 (0.30–1.79)	0.488	0.69 (0.29–1.65)	0.405
		Asian	77%	0.35 (0.07–1.74)	0.200	0.48 (0.10–2.31)	0.358
	I don’t feel embarrassed to go	Overall	42%				
		White	36%	Ref		Ref	
		Black	47%	1.61 (0.74–3.53)	0.232	1.50 (0.63–3.56)	0.356
		Latino	47%	1.62 (0.81–3.27)	0.175	1.70 (0.88–3.30)	0.114
		Asian	63%	3.41 (0.72–16.10)	0.121	4.00 (0.78–20.50)	0.096
	I feel welcome	Overall	89%				
		White	90%	Ref		Ref	
		Black	87%	0.76 (0.27–2.10)	0.590	1.27 (0.46–3.56)	0.645
		Latino	84%	0.60 (0.26–1.39)	0.230	0.88 (0.28–2.81)	0.834
		Asian	99%	7.34 (0.85–63.30)	0.070	22.50 (2.15–236.00)	**0.009**
Pantry Satisfaction	The pantry does not run out of food	Overall	52%				
		White	55%	Ref		Ref	
		Black	69%	1.85 (0.82–4.17)	0.139	1.73 (0.70–4.28)	0.232
		Latino	40%	0.55 (0.27–1.09)	0.086	0.51 (0.25–1.04)	0.066
		Asian	41%	0.31 (0.08–1.14)	0.078	0.34 (0.09–1.29)	0.112
	The hours are convenient	Overall	74%				
		White	77%	Ref		Ref	
		Black	65%	0.54 (0.23–1.27)	0.156	0.61 (0.25–1.52)	0.290
		Latino	74%	0.83 (0.41–1.69)	0.605	0.86 (0.41–1.79)	0.680
		Asian	72%	0.87 (0.15–5.09)	0.878	1.76 (0.29–10.70)	0.536
	The lines/wait times are not long	Overall	38%				
		White	39%	Ref		Ref	
		Black	39%	1.00 (0.44–2.27)	0.999	1.00 (0.45–2.21)	0.996
		Latino	38%	0.97 (0.48–1.98)	0.937	0.79 (0.38–1.64)	0.528
		Asian	31%	0.85 (0.16–4.37)	0.841	1.13 (0.18–7.33)	0.897
	They don’t limit how often we can visit	Overall	48%				
		White	47%	Ref		Ref	
		Black	58%	1.56 (0.71–3.40)	0.268	1.33 (0.58–3.05)	0.503
		Latino	48%	1.05 (0.52–2.13)	0.894	0.72 (0.35–1.49)	0.380
		Asian	43%	0.56 (0.14–2.20)	0.401	0.86 (0.21–3.50)	0.829
	I would recommend to others	Overall	89%				
		White	93%	Ref		Ref	
		Black	91%	0.71 (0.21–2.44)	0.582	1.07 (0.25–4.60)	0.923
		Latino	79%	0.28 (0.09–0.81)	**0.020**	0.29 (0.10–0.88)	**0.029**
		Asian	82%	0.23 (0.03–1.89)	0.171	0.41 (0.05–3.36)	0.406
Food Satisfaction	Food has been helpful	Overall	85%				
		White	87%	Ref		Ref	
		Black	82%	0.69 (0.26–1.86)	0.467	1.05 (0.34–3.25)	0.928
		Latino	84%	0.80 (0.28–2.34)	0.688	1.87 (0.57–6.14)	0.299
		Asian	75%	0.46 (0.06–3.40)	0.444	0.52 (0.06–4.73)	0.558
	Food that household likes to eat	Overall	77%				
		White	77%	Ref		Ref	
		Black	78%	1.06 (0.42–2.72)	0.898	1.75 (0.64–4.79)	0.272
		Latino	74%	0.83 (0.35–1.98)	0.679	1.45 (0.58–3.60)	0.425
		Asian	79%	0.92 (0.13–6.64)	0.932	0.97 (0.08–11.20)	0.979
	Food is good quality	Overall	75%				
		White	76%	Ref		Ref	
		Black	77%	1.06 (0.42–2.64)	0.906	1.88 (0.72–4.91)	0.199
		Latino	67%	0.62 (0.27–1.42)	0.262	1.07 (0.47–2.45)	0.870
		Asian	84%	5.94 (1.27–27.70)	**0.024**	8.85 (1.45–53.90)	**0.018**
	Provides enough food	Overall	66%				
		White	67%	Ref		Ref	
		Black	68%	1.03 (0.45–2.37)	0.947	0.98 (0.37–2.57)	0.960
		Latino	64%	0.87 (0.42–1.82)	0.712	0.85 (0.41–1.76)	0.654
		Asian	59%	1.16 (0.23–6.00)	0.857	1.61 (0.34–7.64)	0.549
	I know how to prepare the foods	Overall	79%				
		White	84%	Ref		Ref	
		Black	89%	1.53 (0.53–4.41)	0.426	2.77 (0.93–8.26)	0.068
		Latino	64%	0.34 (0.15–0.81)	**0.015**	0.83 (0.36–1.93)	0.665
		Asian	73%	0.41 (0.07–2.22)	0.298	0.47 (0.08–2.91)	0.417

Notes: The sample is restricted to those who reported using a food pantry during the COVID-19 pandemic. Models were adjusted for income, presence of children in household, gender, age, and education. Odds ratios with a *p*-value < 0.05 are bolded. ^1^ Sample sizes: Overall = 520; White = 359; Black = 60; Latino = 85; Asian = 16; ^2^ % Agree is defined as the percentage of those selecting “Strongly Agree” or “Agree”.

## Data Availability

Data are contained within the article.

## Appendix A

**Table A1:** Demographic characteristics in the weighted and unweighted sample compared to demographic distribution in Massachusetts, *n* = 2928.

Variable	Category	Unweighted %	Weighted %	Massachusetts (ACS) %
Gender	Female	68.8	52.3	52.1
	Male	31.2	47.7	47.9
Age group, years	18–34	40.3	29.8	30.5
	35–54	34.9	32.4	32.4
	55–64	13.3	17.3	16.9
	65+	11.4	20.5	20.2
Race/ethnicity	Non-Latino White	74.6	74.9	74.2
	Non-Latino Black	6.8	6.5	6.6
	Non-Latino Asian	5.1	6.4	6.6
	Latino	10.0	9.9	10.2
	Other or Multiracial	3.5	2.4	2.4
Educational attainment	High school or less	3.5	8.7	9.3
	High school graduate (including GED)	22.2	24.3	24.6
	Some college (no degree)	21.5	18.8	18.7
	Associate degree/technical school/apprenticeship	11.3	7.2	7.1
	Bachelor’s degree	26.2	23.5	23.1
	Graduate degree	15.3	17.6	17.3
Household income	<$10,000	8.6	3.2	3.6
	$10,000 to $24,999	15.1	7.8	7.9
	$25,000 to $49,999	25.4	13.8	14.0
	$50,000 to $74,999	17.8	14.0	13.9
	$75,000 to $99,999	12.0	12.9	12.7
	$100,000 to $149,999	13.6	21.1	20.7
	$150,000 to $199,999	4.4	12.1	11.7
	≥$200,000	3.0	15.2	15.4
Region	Western (Berkshire, Franklin, Hampshire, Hampden)	14.9	12.3	12.2
	Central (Worcester)	14.3	11.9	11.9
	Boston (Suffolk, Norfolk)	20.9	22.2	22.2
	Northeast (Essex, Middlesex)	31.2	34.6	34.6
	Southeast (Bristol, Plymouth, Barnstable, Dukes, Nantucket)	18.6	19.1	19.2
Children in household	Yes	37.2	37.5	31.9

ACS = American Community Survey.
